# Three Cases of Non-islet Cell Tumor Hypoglycemia Highlighting Efficacy of Glucocorticoid Treatment

**DOI:** 10.1210/jcemcr/luad045

**Published:** 2023-07-13

**Authors:** Kimberly Voon, Aaron Simpson, Peter Gerard Fegan, John P Walsh

**Affiliations:** Department of Endocrinology and Diabetes, Sir Charles Gairdner Hospital, Nedlands, WA 6009, Australia; Department of Clinical Biochemistry, PathWest Laboratory, Queen Elizabeth II Medical Centre, Perth, WA 6009, Australia; Department of Clinical Biochemistry, Clinipath Pathology, Osborne Park, WA 6017, Australia; Department of Endocrinology and Diabetes, Fiona Stanley Hospital, Murdoch, WA 6150, Australia; Medical School, Curtin University, Perth, WA 6102, Australia; Department of Endocrinology and Diabetes, Sir Charles Gairdner Hospital, Nedlands, WA 6009, Australia; Medical School, University of Western Australia, Crawley, WA 6009, Australia

**Keywords:** hypoglycemia, tumor, insulin-like growth factor 2, non-islet cell tumor hypoglycemia, glucocorticoids

## Abstract

Non-islet cell tumor hypoglycemia (NICTH) is a rarely encountered cause of hypoglycemia. It is most often caused by tumor secretion of precursor insulin-like growth factor-2 (IGF-2) which, in high concentrations, binds to insulin receptors exerting insulin-like metabolic effects. It is often associated with mesenchymal and hepatic tumors. We describe 3 cases of NICTH: a 60-year-old man with an unresectable pelvic sarcoma and two women ages 43 and 57 with metastatic hemangiopericytoma. Biochemical assessment identified hypoglycemia associated with suppressed insulin, c-peptide, and beta-hydroxybutyrate levels. Each patient was treated with oral glucocorticoids, which effectively prevented recurrence of hypoglycemia and this effect was sustained long-term. These cases highlight a rarely encountered but important cause of hypoglycemia and demonstrate the long-term efficacy of glucocorticoid treatment in preventing hypoglycemia in cases of NICTH related to surgically unresectable tumors.

## Introduction

Non-islet-cell tumor hypoglycemia (NICTH) is a rare paraneoplastic phenomenon associated with a variety of malignancies including mesenchymal and hepatic tumors [1]. Due to its rarity, it is infrequently encountered by endocrinologists. Due to the risk of dangerous hypoglycemia, it requires prompt recognition so as not to delay treatment. Hypoglycemia is caused by tumor secretion of precursor insulin-like growth factor (IGF)-2, which in large concentrations induces downstream insulin-like metabolic effects [2]. NICTH is thus characterized by the biochemical finding of hypoinsulinemic hypoglycemia [3]. Different management strategies are reported in the literature with complete surgical resection being the most effective long-term treatment option [4]. In the setting of an unresectable tumor burden, a number of different medical therapies have been reported, including glucocorticoid use.

## Case Presentations

 

## Case 1

A 60-year-old male was brought in by ambulance to the emergency department following a collapse at home. Capillary glucose performed by paramedics on scene was found to be unrecordably low. In retrospect, he recalled a three-week history of early morning sweats and tremors. His past medical history was significant for hypertension, hepatitis B, and a pelvic sarcoma. His medications included paracetamol, tramadol, telmisartan, and amlodipine. The sarcoma was diagnosed five years previously and surgically treated, with recurrence three years later. It had been deemed inoperable on recurrence, and the patient had declined chemotherapy.

## Case 2

A 43-year-old health professional with a background of metastatic anaplastic hemangiopericytoma undergoing palliative radiotherapy was found unresponsive in bed by her husband. Capillary glucose performed by paramedics was unrecordably low. She was treated with oral glucose and elected not to attend the emergency department. Her regular medications included sodium valproate and doxycycline. Following the event, she continued home capillary blood glucose level monitoring and noted recurrent hypoglycemic events, with blood glucose levels <2 mmol/L (<36 mg/dL) requiring four hourly meals to mitigate this.

## Case 3

A 57-year-old female was brought in by ambulance to the emergency department following a witnessed seizure at home. Measurement of capillary glucose by attending paramedics was 1.6 mmol/L (28 mg/dL). Her medical history was significant for metastatic hemangiopericytoma complicated by spinal cord compression (for which she was receiving radiotherapy), congestive cardiac failure, and stroke. Her regular medications included levetiracetam, citalopram, furosemide, aspirin, folic acid, bisoprolol, ramipril, and oxycodone.

## Diagnostic Assessment

Patients 1 and 3 were assessed as inpatients. Symptoms of hypoglycemia with demonstrated hypoglycemia on capillary glucose monitoring occurred spontaneously at 1 Am and 10:45 Pm, respectively. Patient 2 attended a pathology collection center as an outpatient during a symptomatic episode at 3 Pm, with demonstrated hypoglycemia on capillary glucose testing. In all cases, venous blood was drawn for laboratory studies before administration of carbohydrate. All patients satisfied Whipple's triad [5], and further laboratory assessment of all three patients subsequently demonstrated hypoinsulinemic hypoglycemia with suppressed beta-hydroxybutyrate confirming the diagnosis of NICTH (see Table 1).

**Table 1.** Relevant biochemistry results

	Patient 1	Patient 2	Patient 3	Reference range
Plasma glucose	2.4 mmol/L (43.2 mg/dL)	1.5 mmol/L (27 mg/dL)	1.6 mmol/L (28.8 mg/dL)	3-5 mmol/L (54-90 mg/dL)
C-peptide	<0.05 nmol/L (<0.02 ng/mL)	<0.05 nmol/L (<0.02 ng/mL)	<0.05 nmol/L (<0.02 ng/mL)	0.2-0.9 nmol/L (0.08-0.4 ng/mL)
Insulin	<1 mU/L (<7 pmol/L)	<1 mU/L (<7 pmol/L)	<1 mU/L (<7 pmol/L)	<12 mU/L (83 pmol/L)
Beta hydroxybutyrate	<0.1 mmol/L (<1.8 mg/dL)	<0.1 mmol/L (<1.8 mg/dL)	<0.1 mmol/L (<1.8 mg/dL)	<0.4 mmol/L (<7.2 mg/dL)
Cortisol	410 nmol/L (14.8 µg/dL)	420 nmol/L (15.2 µg/dL)	369 nmol/L (13.4 µg/dL)	150-700 nmol/L (5.4-25 µg/dL)
IGF-1	28 µg/L (3.6 nmol/L)	<25 µg/L (<3.3 nmol/L)	41 µg/L (5.4 nmol/L)	80-327 µg/L (10-40 nmol/L)
Growth hormone	0.5 µg/L	1.2 µg/L	0.9 µg/L	<3.3 µg/L

Abbreviations: IGF-1, insulin-like growth factor 1.

## Treatment

On confirmation of NICTH diagnosis, all patients were commenced on oral glucocorticoids. Patients 1 and 2 were treated with dexamethasone 4 mg daily, chosen for its long duration of action as both patients experienced recurrent symptomatic overnight hypoglycemia. The dose was selected pragmatically, based on the limited literature available regarding effective glucocorticoid doses in this condition (prednisolone 15-60 mg daily or equivalent). Patient 3 received prednisolone 50 mg daily, with this medication selected as it was part of a planned chemotherapy regimen.

## Outcome and Follow-up

All patients had complete resolution of hypoglycemia with glucocorticoid treatment. Following diagnosis, patient 1 was referred to medical and radiation oncology for further discussion of treatment options. While he again declined chemotherapy, he subsequently completed a treatment course of radiotherapy followed by targeted radioligand therapy which, at the time of writing (one year following diagnosis of NICTH), has resulted in good tumor burden response. While weaning was initially planned, dexamethasone was continued at a dose of 4 mg by his palliative care team for the concurrent management of pain throughout radiotherapy treatment. Following the completion of radiotherapy and evidence of tumor burden response, dexamethasone was weaned over a period of two months supervised by his palliative care physician. Patient 1 monitored his blood glucose levels for two weeks following cessation of dexamethasone with no further hypoglycemia. He remains asymptomatic of hypoglycemia at the time of writing. Patient 2 initially weaned dexamethasone to 1.5 mg daily (required for eligibility for a clinical trial of immunotherapy); however, this resulted in breakthrough hypoglycemia. Despite further cycles of chemotherapy, disease progression prevented any further attempts at steroid weaning, and she was maintained on 4 mg dexamethasone until she succumbed to her disease 6 months after NICTH diagnosis. Patient 3 was titrated over several months to prednisolone 37.5 mg and subsequently 25 mg daily. She continued to receive further cycles of chemotherapy during this time; however, further titration of glucocorticoids was not attempted due to progression of disease. She subsequently succumbed to the disease two years after diagnosis of NICTH. All patients tolerated glucocorticoids with no major adverse effects. Due to the efficacy of glucocorticoid therapy in mitigating hypoglycemia, no other nonglucocorticoid medical therapies were trialed for NICTH management.

## Discussion

Hypoglycemia is an uncommon phenomenon outside of the diabetic population. In this cohort, hypoglycemia often stems from hyperinsulinism via endogenous or exogenous mechanisms. It is rarely associated with hypoinsulinemia. NICTH is a rare paraneoplastic phenomenon initially described in the 1980s [3]. While it can be caused by a wide range of malignancies, it is most often associated with tumors of epithelial cell or mesenchymal origin [1]. The most common epithelial tumor associated with NICTH is hepatocellular carcinoma. Mesenchymal tumors more frequently associated with NICTH include fibrosarcoma, mesothelioma, and hemangiopericytoma. These tumors are commonly large and usually originate in the thorax. In some cases, hypoglycemia precedes the tumor diagnosis. More commonly, however, and as seen in our cases, it occurs following diagnosis of the malignancy [3]. Given the increasing incidence of malignancies, it is expected that the number of reported cases of NICTH will continue to increase.

NICTH is caused by excessive tumor secretion of precursor IGF-2 molecules (big IGF-2). IGF-2 is a single chain polypeptide (7.5 kDa) similar in structure to IGF-1 and proinsulin [2, 4]. It is expressed in the liver, and it functions primarily for cell proliferation and apoptosis during fetal and neonatal development. However, it is present in constant serum concentrations in adulthood [6]. In normal circumstances, up to 80% of IGF-2 is bound to IGF-big protein 3 (IGF-BP3) and the acid-labile subunit (ALS) forming a large ternary complex (approximately 150 kDa), which, due to its large size, does not cross capillary membranes and thus has limited biological activity [2, 4]. A small proportion of IGF-2 is bound only to IGF-BP3 forming a binary complex (approximately 50 kDa), which does cross the capillary membrane and thus is biologically active. In NICTH, abnormal processing of IGF-2 precursors causes high concentrations of big IGF-2 (10-20 kDa) in the plasma. Due to its higher molecular weight, it is unable to bind to ALS and is bound to IGFBP3 only [2, 4]. This 60 kDa structure crosses the capillary membrane resulting in a higher concentration of biologically active IGF-2 [2, 4]. This complex saturates the insulin and IGF receptors exerting insulin-like downstream metabolic effects, resulting in increased glucose uptake in the muscles, decreased glucagon secretion, decreased glycogenolysis, and gluconeogenesis. Via negative feedback mechanisms, IGF-1, growth hormone (GH), and insulin levels decrease. Through a similar mechanism, NICTH from tumor secretion of IGF-1 has been reported in the literature, although it is exceptionally rare [3].

As IGF-2 levels cannot be assayed commercially (and we were unable to measure IGF-2 levels in our cases), the diagnosis of NICTH can be made when hypoinsulinemic hypoglycemia, with a concurrent low C-peptide is demonstrated in the presence of a large tumor [1, 5]. A suppressed beta-hydroxybutyrate is necessary to confirm the presence of an insulin-like effect [5]. Low GH and IGF-1 levels are also a helpful adjunct to diagnosis. In the literature, where IGF-2 assays are available, levels may be normal or high (depending on the assay used), and an IGF-2/IGF-1 molar ratio of >10 helps to confirm the diagnosis of NICTH [4].

Ideally, management of NICTH involves surgical excision of the causative tumor, but this is often not possible. In the literature, partial excision and reduction of tumor burden via localized treatment such as radiotherapy has been found to be effective [4]. This is consistent with evidence of resolution of hypoglycemia in our first case, allowing for cessation of glucocorticoids. In a review of the management of NICTH by Bodnar et al, management with the use of diazoxide or octreotide, as well as systemic treatment with chemotherapy, is often ineffective [4]. In surgically incurable cases, glucocorticoids have been identified to be the most effective management strategy for mitigating hypoglycemia, and, commonly, doses equivalent to prednisone 30 to 60 mg/day have been reported to be effective. Teale et al described eight patients with NICTH who responded well to glucocorticoid treatment, with some responding to prednisone doses as low as 15 mg/day [7]. The proposed mechanism by which glucocorticoids treat NICTH is hypothesized to be decreased circulating levels of big IGF-2 through suppression of tumor secretion and increased clearance via unknown mechanisms. Indeed, Teale et al demonstrated restoration of normal insulin and C-peptide levels, decreased big IGF-2 levels, and increased IGF-1 and ALS levels in a group of four NICTH patients treated with glucocorticoids [8].

Aside from glucocorticoid therapy, treatment with recombinant growth hormone (rGH) has been used successfully to treat hypoglycemia in NICTH. However, this has been documented to require supraphysiological doses of the order of 3 to 12 mg daily [4]. Teale et al demonstrated that in four NICTH patients treated with rGH, its use increased IGFBP3 and ALS levels, which was hypothesized to assist in restoration of the normal ternary complex allowing for normalization of biologically inactive IGF-2 levels [8]. However, they demonstrated that GH treatment did not restore normal insulin and C-peptide levels and, concerningly, significantly increased big IGF-2 levels. Due to the need for supraphysiological doses, its effect on increasing big IGF-2 levels, and its potential to stimulate tumor growth, the use of rGH is thought to be less desirable than glucocorticoids. Combination therapy with glucocorticoids and rGH has been described when monotherapy with either was unsuccessful [4].

## Learning Points

- NICTH is a rare but serious paraneoplastic phenomenon caused by tumor secretion of precursor IGF-2.
- The diagnosis is made on demonstration of hypoinsulinemic hypoglycemia with suppressed beta-hydroxybutyrate levels, usually in the presence of a large tumor burden (IGF-2 assays are not commercially available).
- Reduction of tumor burden can ameliorate or reverse NICTH.
- Hypoglycemia associated with NICTH can be treated with glucocorticoids, and this effect is sustainable long term.
- While high-dose glucocorticoid therapy is usually required initially, the dose can be subsequently titrated to the lowest effective dose in order to mitigate side effects.

## Contributors

K.V., A.S., P.G.F., and J.P.W. were involved in the diagnosis and management of the patients described. K.V. drafted and edited the manuscript. J.P.W., A.S., and P.G.F. contributed to the editing of the manuscript. All authors have read and approved this manuscript.

## Data Availability

Data sharing is not applicable to this article as no datasets were generated or analyzed during the current study.
